# Reoperations after decompression with or without fusion for L4–5 spinal stenosis with or without degenerative spondylolisthesis: a study of 6,532 patients in Swespine, the national Swedish spine register

**DOI:** 10.1080/17453674.2021.1879505

**Published:** 2021-01-28

**Authors:** Anders Joelson, Fredrik Nerelius, Marek Holy, Freyr Gauti Sigmundsson

**Affiliations:** Department of Orthopedics, Örebro University School of Medical Sciences and Örebro University Hospital, Örebro, Sweden

## Abstract

Background and purpose — There are different opinions on how to surgically address lumbar spinal stenosis with concomitant degenerative spondylolisthesis (DS). We investigated reoperation rates at the index and adjacent levels after L4–5 fusion surgery in a large cohort of unselected patients registered in Swespine, the national Swedish spine register.

Patients and methods — 6,532 patients, who underwent surgery for L4–5 spinal stenosis with or without DS between 2007 and 2012, were followed up to 2017 to identify reoperations at the index and adjacent levels. The reoperation rates for decompression and fusion were compared with the reoperation rates for decompression only and for patients with or without DS. Patient-reported outcome data were collected preoperatively, and at 1 and 2 years after surgery and used to evaluate differences in outcome between index operations and reoperations.

Results — For spinal stenosis with DS, the reoperation rate at the index level was 3.0% for decompression and fusion and 6.0% for decompression only. At the adjacent level, the corresponding numbers were 9.7% and 4.2% respectively. For spinal stenosis without DS, the reoperation rate at the index level was 3.7% for decompression and fusion and 6.2% after decompression only. At the adjacent level, the corresponding numbers were 8.1% and 3.8% respectively. For the reoperations at the adjacent level, there was no difference in patient-reported outcome between extended fusion or decompression only.

Interpretation — Single-level lumbar fusion surgery is associated with an increased rate of reoperations at the adjacent level compared with decompression only. When reoperations at the index level are included there is no difference in reoperation rates between fusion and decompression only.

There is an ongoing controversy on how to surgically address lumbar spinal stenosis with and without concomitant degenerative spondylolisthesis (DS). The debate has focused on the durability of the index operation versus accelerated symptomatic degeneration at the adjacent segments. In this debate, durability has been defined as maintenance of clinical benefit without the need for additional intervention (Ghogawala et al. 2017). While decompression without fusion may increase the risk for early index-level reoperations for instability (Ghogawala et al. 2016, Urakawa et al. 2020), fusion surgery might increase the risk of adjacent segment disease (ASD) (Sears et al. 2011, Okuda et al. 2018).

The national Swedish spine register (Swespine) covers 90% of the spine units in Sweden and the follow-up rate is 75–80% (Strömqvist et al. 2013). The Swespine offers possibilities to examine outcome in a large dataset of patients operated on for isolated L4–5 disease, which is the most common clinical scenario within spine surgery. Large register studies may contribute to increased evidence in this area.

Using Swespine data, we investigated reoperation rates at the index and the adjacent levels after L4–5 decompression only or decompression and fusion for spinal stenosis with and without concomitant DS.

## Patients and methods

### Study design

National Register study with prospectively collected data.

### Patients

The Swespine is a national quality register administered by the Swedish Association of Spine Surgeons. Demographic information is registered and follow-up forms are mailed to patients with a prepaid return envelope. Participation is voluntary for both the patients and the participating clinics and can be withdrawn at any time (Strömqvist et al. 2013).

We identified 6,584 patients who underwent surgery for lumbar L4–5 spinal stenosis with or without DS between 2007 and 2012. 52 patients were subject to acute reoperations (e.g., due to postoperative hematomas) and were excluded. The remaining 6,532 patients were followed up between 2007 and 2017 to identify reoperations. The diagnosis spinal stenosis was reported by the operating surgeon. DS was defined as slip > 3 mm on preoperative radiographs. Only patients who underwent surgery at the L4–5 level were included in the study.

### Reoperation rates

In Swespine, index operations and reoperations are registered separately. In order to identify reoperations that were incorrectly classified as index operations, we scanned the register for patients who had multiple operations. Reoperations because of spinal stenosis with or without concomitant DS or disk herniation at the index level (L4–5), the 1st cranial adjacent level (L3–4), the 2nd cranial adjacent level (L2–3), and the 1st caudal adjacent level (L5–S1) were counted. The diagnoses spinal stenosis and disk herniation were reported by the operating surgeon. Only the 1st reoperation was counted. Reoperations at the index level (L4–5) because of implant failure were counted separately.

### Patient-reported outcome measures

Patient-reported outcome measures (PROMs) were recorded preoperatively, and at 1 and 2 years after surgery. We evaluated pain, disability, and health-related quality of life. Numeric rating scales (NRS) were used to assess leg and back pain (range 0–10, 0 being the best). The Oswestry disability index version 2.0 (ODI) (Fairbank and Pynsent 2000) was used to assess disability (range 0–100, 0 being the best and 100 the worst). The EQ5D index (UK tariff) (EuroQol Group 1990, Dolan 1997) was used to assess health-related quality of life (range –0.59 to 1, –0.59 being the worst and 1 the best).

### Statistics

The results are presented as mean (SD). Student’s t-test for unpaired data was used to compare normally distributed data. The Mann–Whitney U-test was used to compare unpaired non-normally distributed data. The chi-square test was used to compare frequencies. A p-value of < 0.05 was considered significant, and 2-tailed tests were used. The 95% confidence interval (CI) for relative risks is calculated as described by Altman (1991).

### Ethics, data sharing, funding, and potential conflicts of interest

The study was approved by the regional ethical review board (registration number: 2020-01505). Data are available from the national Swedish spine register (Swespine) after approval by a Swedish regional ethical review board and approval by the Swespine board. There was no external source of funding for this study. The authors declare no conflicts of interest.

## Results

### Baseline (Table 1)

There were no statistically significant differences in BMI and mean duration of follow-up but there were some minor differences in age and sex distributions.

### Reoperation rates (Figure 1,Table 2)

For spinal stenosis with DS, the reoperation rate at the index level (L4–5) was 3.0% for decompression and fusion and 6.0% for decompression only. At the 1st cranial adjacent level (L3–4), the corresponding numbers were 9.7% and 4.2% respectively. For spinal stenosis without DS, the reoperation rate at the index level (L4–5) was 3.7% for decompression and fusion and 6.2% after decompression only. At the 1st cranial adjacent level (L3–4), the corresponding numbers were 8.1% and 3.8% respectively. There were in total 38 reoperations because of implant related problems (loosening 4, breakage 1, pain because of implant 24, and pseudarthrosis 9).

### Risk factors for additional surgery (Table 3)

There was a minor age difference but no statistically significant difference in BMI, sex, or fusion method for patients with DS who underwent additional surgery at the adjacent level after decompression and fusion compared with the patients that required no additional surgery at the adjacent level.

### Patient-reported outcome measures (Figures 2 and 3)

The 1-year and 2-year response rates for PROMs were 73% and 65% respectively. For all PROMs, there was no statistically significant difference between decompression and fusion and decompression only.

## Discussion

Adjacent segment disease after lumbar spinal fusion surgery has been a topic of concern for many years. The randomized controlled trials (RCTs) of Försth et al. (2016) and Ghogawala et al. (2016) did little to settle the controversy as the results were divergent. We report reoperation rates at adjacent segments for the largest cohort of patients who underwent surgery for lumbar L4–5 spinal stenosis.

We found a statistically significant lower risk for reoperation at the index level in the fusion group compared with the decompression group. When the reoperations at index level were counted together with the reoperations at the adjacent level there were no statistically significant differences in reoperation rates between decompression and fusion and decompression only. Also, Försth et al. (2016) and Ghogawala et al. (2016) found that reoperations at the index level were more common after decompression only while reoperations at adjacent levels were more common after fusion. Our results confirm the results of Radcliff et al. (2013) that lumbar fusion is not associated with increased reoperation rate compared with decompression only.

The overall reoperation rates reported by Sears et al. (2011) and Radcliff et al. (2013) were similar to ours. The RCT of Försth et al. (2016), however, reported higher reoperation rates but that study included multilevel procedures. Sears et al. (2011) found a correlation between the number of levels fused and reoperation rate, which might explain our somewhat lower reoperation rate. Moreover, we excluded 52 acute reoperations, which reduce our reoperation rate. Our lower reoperation rate may also be related to coverage issues of register data.

**Figure 1.**
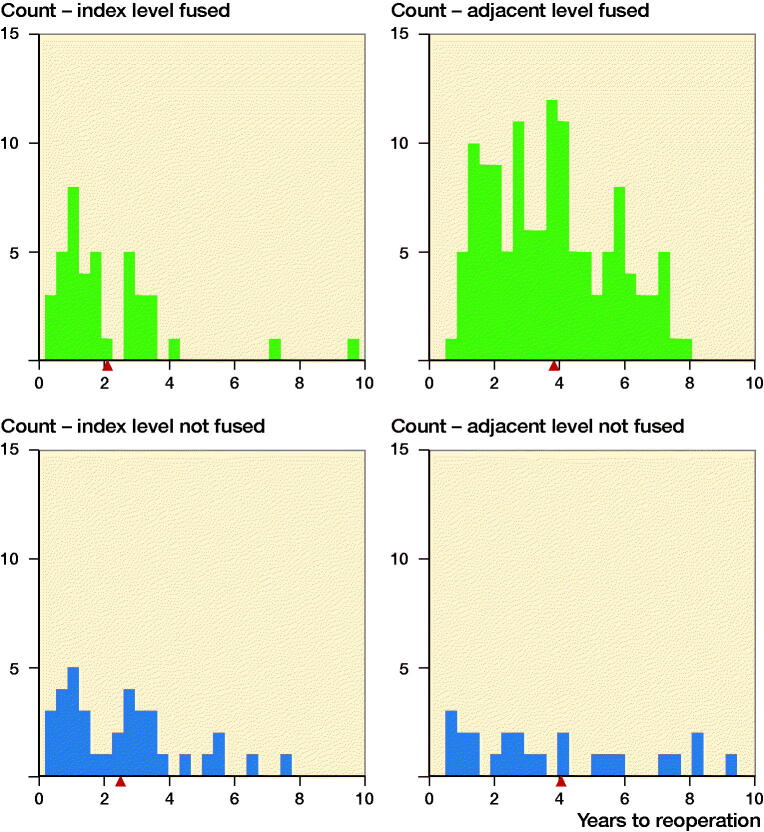
Histogram of time to reoperation at the index level (L4–5) and adjacent level (L3–4) for patients with spinal stenosis and degenerative spondylolisthesis after decompression and fusion or decompression only. 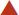 indicates the mean values.

Our data suggests that the times to reoperation were shorter for reoperations at the index level compared with reoperations at the adjacent levels (Figure 1). A possible explanation is that reoperations at the index level may have several causes, e.g., insufficient decompression, instability, or implant failure, that require more urgent intervention while ASD is a more slowly progressing condition.

**Figure 2.**
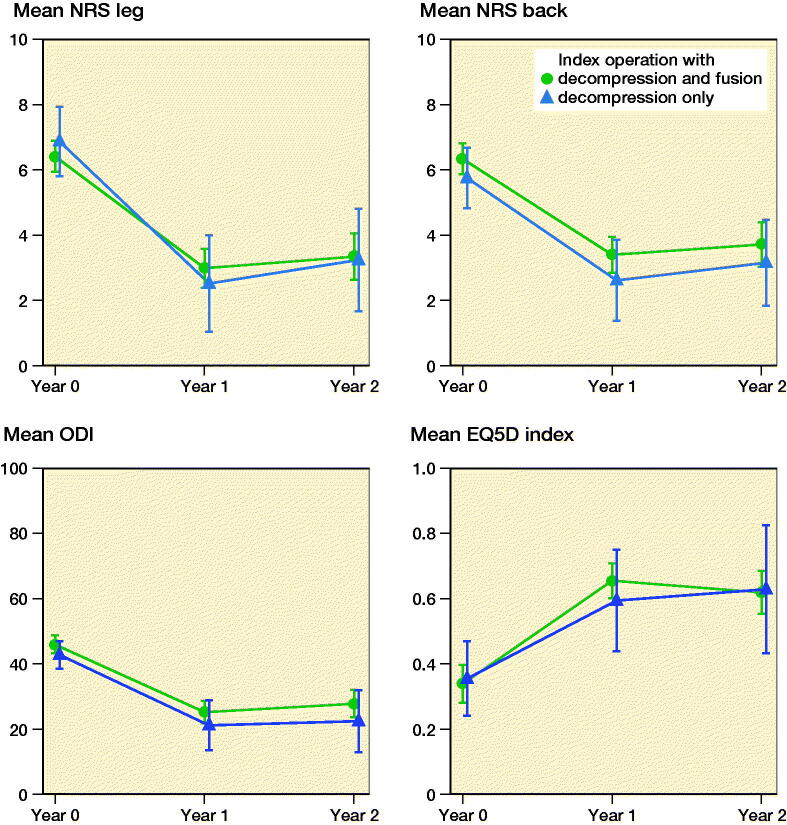
Patient-reported outcome measures for the index operations (L4–5) for patients with L4–5 spinal stenosis and degenerative spondylolisthesis after decompression and fusion or decompression only. Non-responders at the 1- and 2-year follow-up were not excluded. The error bars represent 95% confidence intervals.

The reoperation rate at the 1st caudal level (L5–S1) was low and we agree with Maragkos et al. (2020) that surgeons should refrain from prophylactic procedures at the L5–S1 level when considering a posterior L4–L5 fusion.

For spinal stenosis with DS, the reoperation rate at the 1st adjacent level was 4% for decompression only (Table 2). We agree with Bydon et al. (2016) that this implies that increased rigidity across previously mobile segments introduced by spinal fusion is not the only contributing factor in the development of ASD.

**Table 2.** Results by procedure after L4–5 decompression and fusion or decompression only at the index level (L4–5), 1st cranial adjacent level (L3–4), 2nd cranial adjacent level (L2–3), and 1st caudal adjacent level (L5–S1)

	Stenosis with DS			Stenosis without DS		
	Decompression and fusion	Decom- pression only		Decompression and fusion	Decom- pression only	
Reoperations, n (%)	n = 1,338	n = 597	RR (95% CI)	n = 481	n = 4,116	RR (95% CI)
Index level						
excluding implant failure	11 (0.8)	36 (6.0)	0.1 (0.07–0.3)	9 (1.9)	255 (6.2)	0.3 (0.2–0.6)
including implant failure	40 (3.0)	36 (6.0)	0.5 (0.3–0.8)	18 (3.7)	255 (6.2)	0.6 (0.4–1.0)
Adjacent level						
1st cranial (L3–4)	130 (9.7)	25 (4.2)	2.3 (1.5–3.5)	39 (8.1)	155 (3.8)	2.2 (1.5–3.0)
2nd cranial (L2–3)	39 (2.9)	10 (1.7)	1.7 (0.9–3.5)	7 (1.5)	57 (1.4)	1.1 (0.5–2.3)
1st caudal (L5–S1)	7 (0.5)	9 (1.5)	0.3 (0.1–0.9)	5 (1.0)	73 (1.8)	0.6 (0.2–1.4)
Total	216 (16.1)	80 (13.4)	1.2 (0.9–1.5)	69 (14.3)	540 (13.1)	1.1 (0.9–1.4)

**Table 3.** Characteristics of patients with degenerative spondylolisthesis who underwent additional surgery at the adjacent level after L4–5 decompression and fusion compared with patients who required no additional surgery at the adjacent level

	Reoperation at 1st adjacent level (L3–4)		
	Yes	No	
Parameter	n = 130	n = 1,208	p-value
Mean age (SD)	63 (8.9)	65 (9.5)	< 0.01
BMI (SD)	27 (4.0)	27 (4.6)	0.8
Women, n (%)	103 (80)	924 (76.5)	0.5
Fusion method, n (%)			0.2
PLF	112 (86)	1101 (91.1)	
PLIF	8 (6)	47 (3.9)	
TLIF	10 (8)	60 (5.0)	

**Figure 3.**
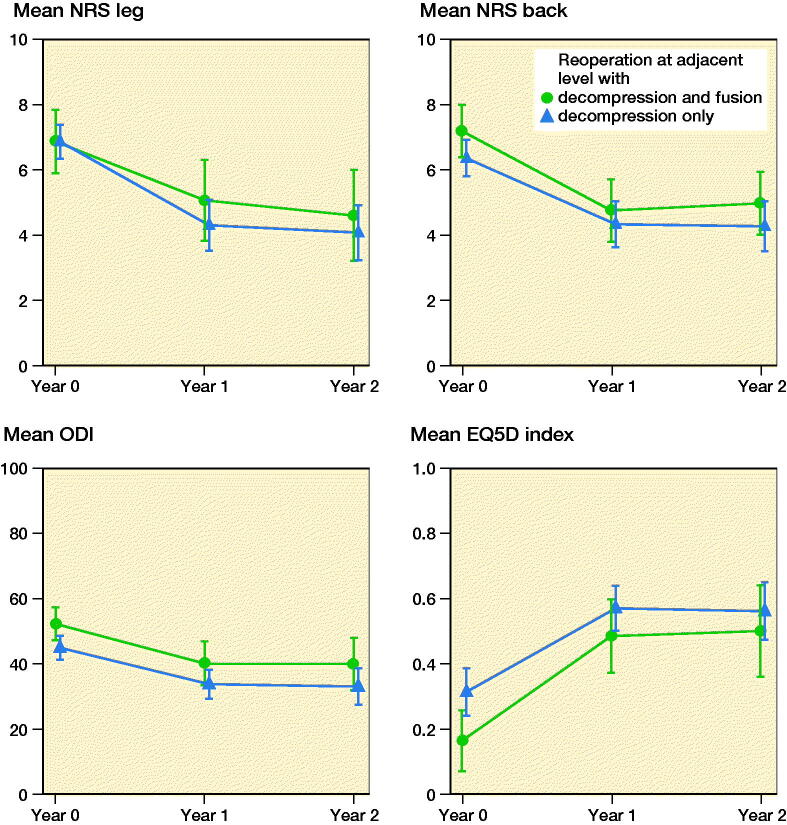
Patient-reported outcome measures for the reoperations at the 1st adjacent level (L3–4) with decompression and extended fusion (n = 41) or decompression only (n = 79) for patients who underwent decompression and fusion for L4–5 spinal stenosis with degenerative spondylolisthesis. Non-responders at the 1- and 2-year follow-up were not excluded. The error bars represent 95% confidence intervals.

The improvements in PROMs after revision surgery for ASD were similar to the minimal important change values reported by Parai et al. (2020) (Figure 3). Also Park et al. (2004) reported that surgery for ASD had relatively modest outcomes. This is an important finding in the context of patient information and shared decision-making. Patients should be informed that the results of a reoperation might be inferior in comparison with the index operation.

We found no statistically significant difference in sex or BMI for patients who required additional surgery at the adjacent level compared with the patients who required no additional surgery. Several authors have reported that sex is not associated with the development of ASD (Lee et al. 2014, Heo et al. 2015, Ou et al. 2015). Contrary to our findings, Ou et al. (2015) found an association between BMI and ASD development. That study included only 13 cases of ASD and used MRI and clinical findings (i.e., not reoperations) to define ASD.

Sears et al. (2011), Lee et al. (2014) and Heo et al. (2015) found that increasing age was a risk factor for ASD requiring further surgery. In contrast, Radcliff et al. (2013) found no association between age and reoperation rate. Although statistically significant, the difference in mean age between the groups of our study was small. If age is an important factor in the development of ASD, it might be more correct to assess biological age or frailty rather than chronological age. Furthermore, a possible confounding factor when studying age and surgery is that younger patients may be more likely to be selected for additional surgery than older patients. This might be one part of the explanation why the patients in the reoperation group of our study are younger than the patients who did not require additional surgery.

We could not find any statistically significant association between type of fusion and additional operations at the adjacent level. In contrast, Lee et al. (2014) and Heo et al. (2015) found associations between fusion types and development of ASD, but their findings were inconsistent. Lee et al. (2014) found that interbody fusion showed a higher incidence of ASD requiring surgery than posterolateral fusion while Heo et al. (2015) reported that posterolateral fusion showed a lower survival rate compared with interbody fusion. The importance of type of surgery on the development of ASD remains to be established.

Our study has several limitations. We recognize the limitations of a register study, e.g., different implants, surgical techniques, postoperative regimen, and selection bias. In this register study, no radiographs were available for analysis. Furthermore, higher response rates for the PROMs would have strengthened our results but studies following non-responders have revealed similar results in terms of the PROMs (Solberg et al. 2011). Our primary outcome variable, the reoperation rate, was of course not affected by the missing PROMs. Moreover, to present our data in a simple and transparent way we did not adjust the reoperation rates and the PROMs for differences in baseline data by using advanced statistical methods, e.g., regression analysis or propensity score matching. Given the diverging results of previous studies (Sears et al. 2011, Radcliff et al. 2013) concerning age as a confounding factor in relation to ASD, we find unlikely that the minor age differences in our groups have any substantial impact on the results.

In conclusion, single-level lumbar fusion surgery at the L4–5 level is associated with an increased rate of reoperations at the proximal adjacent level compared with decompression only. However, when reoperations on the index level are included there is no difference in reoperation rates between fusion and decompression only. Addressing the index segment for durable outcome should be the primary objective of surgery. This can be achieved with decompression or decompression and fusion with similar risk for additional surgery in selected cases.

**Table 1.** Baseline demographics by procedure after L4–5 decompression and fusion or decompression only

	Stenosis with DS			Stenosis without DS		
	Decompression and fusion	Decompression only		Decompression and fusion	Decompression only	
Parameter	n = 1,338	n = 597	p-value	n = 481	n = 4,116	p-value
Mean age (SD)	65 (9.1)	69 (9.9)	< 0.01	60 (10.6)	65 (11.4)	< 0.01
BMI (SD)	27 (4.5)	27 (4.1)	0.02	27 (4.1)	27 (4.1)	0.3
Women, n (%)	1,027 (77)	407 (68)	< 0.01	266 (55)	2,097 (51)	0.07
Years of follow-up (SD)	7.8 (1.6)	7.9 (1.8)	0.4	7.7 (1.6)	7.8 (1.7)	0.5
